# Processing Chain for Localization of Magnetoelectric Sensors in Real Time

**DOI:** 10.3390/s21165675

**Published:** 2021-08-23

**Authors:** Christin Bald, Gerhard Schmidt

**Affiliations:** Digital Signal Processing and System Theory, Institute of Electrical Engineering and Information Technology, Faculty of Engineering, Kiel University, 24143 Kiel, Germany; cbal@tf.uni-kiel.de

**Keywords:** localization, magnetoelectric sensors, real time, pose estimation

## Abstract

The knowledge of the exact position and orientation of a sensor with respect to a source (distribution) is essential for the correct solution of inverse problems. Especially when measuring with magnetic field sensors, the positions and orientations of the sensors are not always fixed during measurements. In this study, we present a processing chain for the localization of magnetic field sensors in real time. This includes preprocessing steps, such as equalizing and matched filtering, an iterative localization approach, and postprocessing steps for smoothing the localization outcomes over time. We show the efficiency of this localization pipeline using an exchange bias magnetoelectric sensor. For the proof of principle, the potential of the proposed algorithm performing the localization in the two-dimensional space is investigated. Nevertheless, the algorithm can be easily extended to the three-dimensional space. Using the proposed pipeline, we achieve average localization errors between 1.12 cm and 6.90 cm in a localization area of size 50cm×50cm.

## 1. Introduction

For the correct solution of inverse problems, such as source reconstruction of biomedical sources, it is essential to know the exact position and orientation of the measuring sensors with respect to the source besides measuring the biomedical signals. Especially in magnetic measurements the sensors do not necessarily have a fixed position and orientation. Thus, a determination of the position and orientation at the beginning of a measurement is not sufficient. Much more desirable is a continuous estimation of the sensor’s position and orientation simultaneously with the measurement [1,2].

Magnetic tracking systems are used in many applications, e.g., in indoor positioning systems [3,4] or to locate medical devices inside the body [5,6]. Moreover, magnetic localization approaches are used to determine the position of the subject relative to the sensor array in biomagnetic measurements. A procedure for determining the subject relative to the measuring sensor array, either once at the beginning or also simultaneously with the measurement, was presented in [7]. In [1,2] a method for determining the positions of the individual sensors in a flexible on-scalp MEG system relative to the subject was investigated, which can also be applied during measurement.

Until now, mainly SQUIDs (Super Conducting Quantum Interference Devices) [8] and recently OPMs (Optically Pumped Magnetometers) [9,10] are used for the measurement of biomagnetic signals. Unfortunately, these sensors require a magnetically well shielded environment and are therefore inconvenient in operation. Magnetoelectric sensors, on the other hand, do not require any shielding, no expensive cooling system, and are also very small in size, which makes them ideal for array applications. The sensors are composed of a magnetostrictive and a piezoelectric layer and use the resonant structure of a cantilever [11]. Detection limits in the sub-nT regime have been reached recently [12,13,14,15] using modulation techniques such as the ΔE-effect [16] for the detection of low-frequency magnetic fields. Consequently, magnetoelectric sensors could be a promising alternative for biomagnetic field measurements in the near future [17].

**Figure 1:**
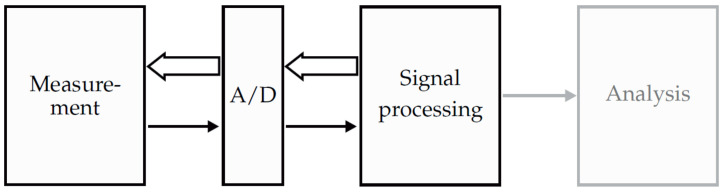
General overview of a medical system operating with magnetic sensors. The measurements are performed simultaneously with localizing the sensors. After transforming the signals into the digital domain, the signals are processed and analyzed. Since the analysis of the measured magnetic signals is not in the focus of this contribution, the corresponding box is depicted in gray.

Since the feasibility of measurements with simultaneous localization using magnetoelectric sensors has been shown in a previous work [15], this work will focus on the processing chain for an enhanced localization of magnetoelectric sensors in real time. However, by adapting the coil excitation signals the method can be used for arbitrary magnetic field sensors. This contribution will step through the general processing overview shown in Figure 1.

In Section 2, the magnetoelectric sensor used in this paper will be presented and characterized. After explaining the so-called forward problem in Section 3, the real-time localization approach will be presented in Section 4. The presented localization processing chain will be verified by measurements presented in Section 5. The paper closes with a conclusion and an outlook in Section 6.

## 2. Magnetoelectric Sensor

**Figure 2:**
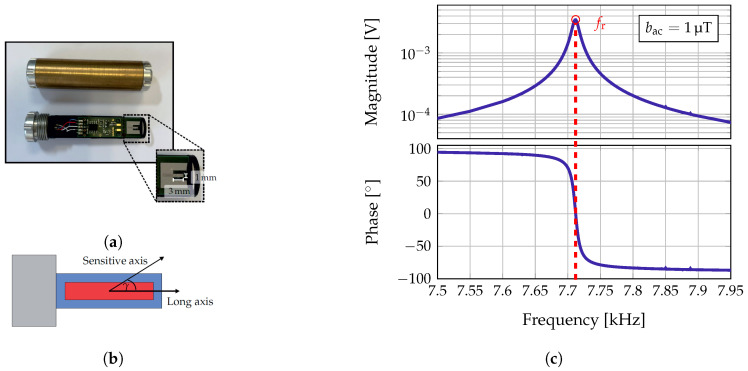
(**a**) Exchange bias ΔE-effect sensor used in this study. The sensor is based on a cantilever of size 3 mm × 1 mm. The cantilever is placed on a printed circuit board and connected to a low-noise JFET charge amplifier [19]. The sensor is encapsulated by a brass cylinder for electrical shielding and mechanical protection. (**b**) Visualization of the relationship between the sensitive and the long axis of the sensor. (**c**) Magnitude and phase response of the sensor in the first bending mode applying a magnetic field of *b*_ac_ = 1 μT. The sensor has a resonance frequency of *f*_r_ = 7.712 kHz and a bandwidth of *bw*_−3 dB_ = 10.2 Hz.

For the proof of principle an exchange bias ΔE-effect sensor as depicted in Figure 2a will be used in this contribution. A sensor of the same type has already been used in a previous work [15]. The sensor is based on a polysilicon cantilever with a size of 1 mm width, 3 mm length, and 50 μm thickness. The cantilever is covered by a 4 μm thick magnetic multilayer (20 × Ta/Cu/MnIr/FeCoSiB) and a 2 μm thick piezoelectric layer (AlN). Further details about the fabrication process of the sensor can be found in [15]. The magnetic multilayer consists of ferromagnetic and antiferromagnetic layers, which ensure the self-biasing of the sensor and thus lead to a shift of the magnetization curve of the sensor [18]. Hence, the sensor can be operated without applying an external bias field, which is especially favorable regarding array applications. The sensor is connected to a low-noise JFET charge amplifier [19] and placed on a printed circuit board. The whole sensor system is encapsulated in a 2.1 mm thick brass cylinder for electrical shielding.

As shown in [15], the localization of the magnetoelectric sensor can be performed simultaneously with a measurement without loss of information or degradation of the signals. The first bending mode was used to localize the sensor, while an artificial heart signal was measured in the second mode using the ΔE-effect. Hence, also in this contribution only frequencies around the first bending mode will be used for the transmission of the localization signals. The amplitude and phase response around the first bending mode of the magnetoelectric sensor used are shown in Figure 2c. The characterization measurements have been performed in a magnetically, electrically, and acoustically shielded environment [11]. The magnitude and phase response of the sensor have been measured applying a magnetic field of $b_{\mathrm{ac}}=1$ μT on the sensor’s long axis. The performance of the sensor can be determined as described in [20]. The sensor has a resonance frequency of $f_{r}=7.712\;\mathrm{kHz}$ and a −3 dB bandwidth of $bw_{-3\mathrm{dB}}=10.2\;\mathrm{Hz}$. Since the brass cylinder acts as a low-pass filter with a cut-off frequency of approximately $f_{c}\approx 1.5\;\mathrm{kHz}$ [15], the sensor’s performance will be improved when removing the brass cylinder. Nevertheless, the encapsulation is necessary for electrical shielding and acts as a mechanical protection. Moreover, the limit-of-detection in the first bending mode with brass cylinder is still sufficient, because the coils can simply emit higher field amplitudes. The maximum sensitivity of the sensor is reached when a magnetic field is applied on the sensitive axis of the sensor. However, the sensitive axis of the sensor is not necessarily equal to the long axis of the sensor. There can be a tilt γ between these two axes [15,21], which is visualized in Figure 2b.

## 3. Forward Problem

**Figure 3:**
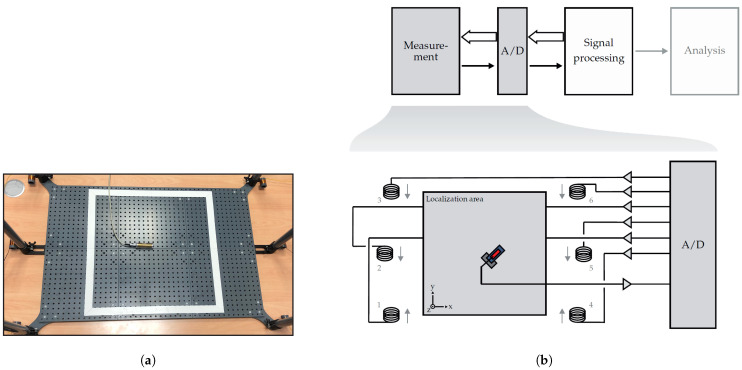
Real (**a**) and schematic (**b**) measurement setup for the localization of magnetoelectric sensors. The coils are placed outside of the localization area and transmit orthogonal signals, which are measured by the sensor. The localization area (box bounded by white stripes in (**a**)) is of size 50 cm × 50 cm.

For the localization of the magnetoelectric sensor, coils are placed outside the localization area as shown in Figure 3. If the distance between the sensor and the coil is large enough, the magnetic field of the coil *i* at the sensor position ${\vec{r}}_{s}\left(t\right)$ can be approximated by the field of a magnetic dipole [22]:

(1) $${\vec{b}}_{i}\left(t,{\vec{r}}_{s}\left(t\right)\right)\;=\;\frac{\mu_{0}}{4\pi }\;\frac{3\;{\vec{r}}_{\mathrm{cs},i}\left(t\right)\left({\vec{m}}_{c,i}{\left(t\right)}^{T}\;{\vec{r}}_{\mathrm{cs},i}\left(t\right)\right)-{\vec{m}}_{c,i}\left(t\right){∥{\vec{r}}_{\mathrm{cs},i}\left(t\right)∥}_{2}^{2}}{{∥{\vec{r}}_{\mathrm{cs},i}\left(t\right)∥}_{2}^{5}}$$

**Figure 4:**
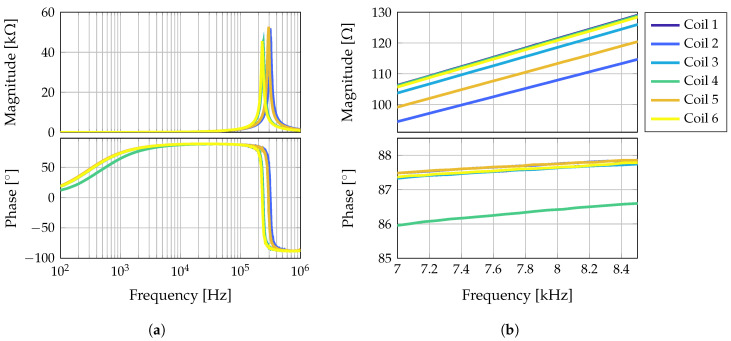
Coil impedances separated into magnitude and phase. In (**a**) the whole spectrum from 100 Hz up to 1 MHz is shown, so that the resonance of the coils can be seen. In (**b**) the frequency range is scaled to the frequency range of the excitation signals.

Here, $\mu_{0}$ is the permeability of vacuum, ${\vec{m}}_{c,i}\left(t\right)$ the magnetic dipole moment of the coil *i*, and ${\vec{r}}_{\mathrm{cs},i}\left(t\right)\;=\;{\vec{r}}_{s}\left(t\right)-{\vec{r}}_{c,i}$ the distance vector between the sensor at position ${\vec{r}}_{s}\left(t\right)$ and the coil *i* at position ${\vec{r}}_{c,i}$. The superscript T denotes the transpose of the vector. The positions of the coils ${\vec{r}}_{c,i}$ are fixed during the measurement and therefore time independent. In this study, $N_{c}=6$ coils have been used. The coils have an effective diameter of 2.6 cm and consist of about 350 turns of enameled copper wire with a wire cross section of 0.13 mm${}^{2}$. The impedances of the six coils, separated into magnitude and phase, are shown in Figure 4.

The signal measured by the sensor

(2) $$u_{\mathrm{in}}\left(t\right)\;=\;h_{s}\left(t\right)*\left({\vec{d}}_{s}^{\;\;T}\left(t\right)\sum_{i=1}^{N_{c}}{\vec{b}}_{i}\left(t,{\vec{r}}_{s}\left(t\right)\right)\right)$$

can be described as a voltage at the output of the sensor system. At the location of the sensor a superposition of the magnetic fields of the coils is present. Due to the directional characteristic of the sensor ${\vec{d}}_{s}\left(t\right)$, only a part of the applied magnetic field is picked up. The conversion of magnetic field into voltage by the sensor system (including the charge amplifier) is described by the impulse response $h_{s}\left(t\right)$. Equation (2) is valid at least for the frequencies around the first bending mode [15]. For simplification, no noise sources are considered here.

## 4. Localization Processing Chain

**Figure 5:**
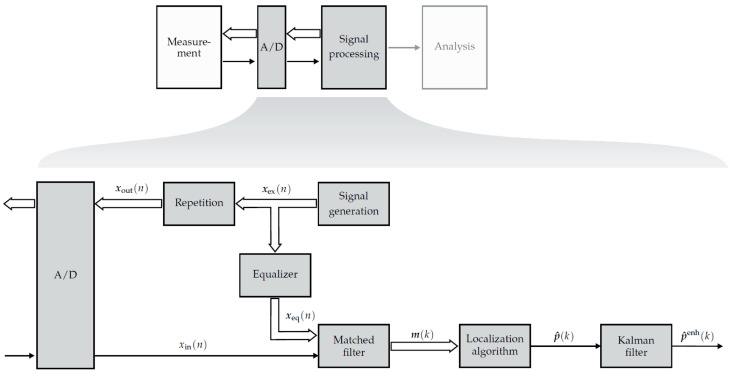
Processing chain for the localization of magnetoelectric sensors in real time. The input signal of the sensor is matched filtered with the equalized coil signals. The matched filter outputs at time lag zero are compared with the lead-field matrix entries. A first order Kalman filter smooths the estimated position-orientation-pairs over time to mitigate possible outliers.

For the estimation of the sensor’s position and orientation an inverse problem must be solved. The processing chain for solving this inverse problem is shown in Figure 5.

For this purpose, the localization area is first divided into a discrete grid containing $N_{p}$ different position-orientation-pairs $P=[p_{1},\ldots ,p_{j},\ldots ,p_{N_{p}}]$, with $p_{j}={\left[{\vec{r}}_{p,j}^{\;\;T},\;{\vec{d}}_{p,j}^{\;\;T}\right]}^{T}$ consisting of a position vector ${\vec{r}}_{p,j}$ (containing x, y, and z components) and an orientation vector ${\vec{d}}_{p,j}$ (directivity described by roll φ, pitch θ, and yaw ψ). The lead-field matrix

(3) $$A=\left[a_{1},\ldots ,a_{j},\ldots ,a_{N_{p}}\right]=\left[\begin{array}{ccccc} a_{11} & \cdots & a_{1j} & \cdots & a_{1N_{p}}\\ \vdots & \ddots & \vdots & \ddots & \vdots \\ a_{i1} & \cdots & a_{ij} & \cdots & a_{iN_{p}}\\ \vdots & \ddots & \vdots & \ddots & \vdots \\ a_{N_{c}1} & \cdots & a_{N_{c}j} & \cdots & a_{N_{c}N_{p}} \end{array}\right]$$

describes the forward problem for the defined position-orientation-pairs in P. That means, the lead-field matrix entry of row *i* and column *j* is defined as

(4) $$a_{ij}\;=\;{\vec{d}}_{p,j}^{\;\;T}\;\frac{3\;{\vec{r}}_{\mathrm{cp},ij}\;\left({\vec{d}}_{c,i}^{\;\;T}\;\;{\vec{r}}_{\mathrm{cp},ij}\right)-{\vec{d}}_{c,i}{∥{\vec{r}}_{\mathrm{cp},ij}∥}_{2}^{2}}{{∥{\vec{r}}_{\mathrm{cp},ij}∥}_{2}^{5}}$$

and thus describes the influence of coil *i* on the sensor, if the sensor would have the position and orientation described by $p_{j}$. The distance vector is defined as ${\vec{r}}_{\mathrm{cp},ij}={\vec{r}}_{p,j}-{\vec{r}}_{c,i}$ and the orientation vector ${\vec{d}}_{p,j}$ can be described by the angles θ and ψ (φ is always zero here) using [23]:

(5) $${\vec{d}}_{p,j}\;=\;\left(\begin{array}{c} \cos(\theta )\cos(\psi )\\ \cos(\theta )\sin(\psi )\\ -\sin(\theta ) \end{array}\right)$$

Equation (4) is a reduced magnetic dipole equation. The prefactor of Equation (1) is neglected and the magnetic dipole moment of the coil is reduced to the orientation of the coil.

### 4.1. Signal Generation and Equalizer

To separate the mixed signals received by the sensor, the signals of the coils must be orthogonal. Two signals $x_{i}\left(n\right)$ and $x_{j}\left(n\right)$ are orthogonal for $n\in \{L_{1},\ldots ,L_{2}\}$, if the following condition

(6) $$\sum_{n=-L_{1}}^{L_{2}}x_{i}\left(n\right)\;x_{j}^{*}\left(n\right)\;=\;\left\{\begin{array}{cc} E_{i}, & i=j\\ 0, & i\neq j \end{array}\right.$$

is fulfilled [24]. Extending this equation to the constant $E_{i}$ being 1, the signals are called orthonormal [24]. This is necessary to extract the individual coil amplitudes from the sensor signal and make them comparable. Different approaches can be used for the generation of orthogonal signals, e.g., using a TDMA (Time Division Multiple Access), an FDMA (Frequency Division Multiple Access) or a CDMA (Code Division Multiple Access) approach [25]. Due to the small bandwidth of the sensor, a TDMA approach is used in this contribution. Thus, the excitation signals

(7) $$x_{\mathrm{ex},i}\left(n\right)\;=\;\cos\left(2\pi f_{r}\left(n-\kappa_{i}\right)\right)\;w\left(n-\kappa_{i}\right)\;\mathrm{with}\;\kappa_{i}=\left(i-1\right)L_{\mathrm{mf}}\;\forall i\;\in \left\{1,\ldots ,N_{c}\right\}$$

are cosine signals at the resonance of the magnetoelectric sensor [15]. The signals are weighted with a Hann window w(n)[26] of length $L_{\mathrm{sig}}$, so that a smoothed in- and out-fading of the signals is ensured. Additionally, the condition $L_{\mathrm{mf}}\geq L_{\mathrm{sig}}$ must be fulfilled. If $L_{\mathrm{mf}}>L_{\mathrm{sig}}$, there is a pausing time between two consecutive coil signals. This is important when considering the impulse response of the sensor. The excitation signals are repeated every $L_{r}=N_{c}L_{\mathrm{mf}}$ samples

(8) $$x_{\mathrm{out},i}\left(n\right)=x_{\mathrm{ex},i}\left(n-\lambda L_{r}\right)\;\mathrm{with}\;\lambda \in Z$$

and transmitted by the coils after D/A conversion. It should be noted that $L_{r}=L_{\mathrm{mf}}$ if an FDMA or a CDMA approach is chosen, because the signals are transmitted simultaneously by all coils.

As can be seen from Equation (1) the magnetic field of a coil is proportional to the driving current. Since the output of the D/A converter is proportional to a voltage, the excitation signals $x_{\mathrm{ex}}\left(n\right)={\left[x_{\mathrm{ex},1}\left(n\right),\ldots ,x_{\mathrm{ex},N_{c}}\left(n\right)\right]}^{T}$ are linearly deformed in amplitude and phase. This can be described by the impulse response of the coil $h_{c,i}\left(n\right)$, denoting the relationship between the voltage and the current of the coil *i*. Additionally, the signals are modified by the impulse response of the magnetoelectric sensor $h_{s}\left(n\right)$, as can be seen from Equation (2). Thus, the signals $x_{\mathrm{ex}}\left(n\right)$ must be equalized either prior to the deformation due to the coil and sensor impulse response or before they are forwarded to the matched filter. In this contribution, the matched filter impulse responses are adapted to match with the modified transmitted signals, so that they are again comparable to the coil signals measured by the sensor. Each coil excitation signal is adjusted individually by the equalizer

(9) $$h_{\mathrm{eq},i}\left(n\right)\;=\;{\hat{g}}_{c,i}\;{\hat{h}}_{c,i}\left(n\right)*{\hat{h}}_{s}\left(n\right)\;,$$

with ${\hat{h}}_{c,i}\left(n\right)$ and ${\hat{h}}_{s}\left(n\right)$ denoting the approximated impulse responses of the coil *i* and the magnetoelectric sensor, respectively. The factor ${\hat{g}}_{c,i}$ describes the influence of other components in the measurement setup. This includes for example different gains of the individual coil amplifier channels and different conversion factors of the coils from current to magnetic field. This is, e.g., due to variances in the number of windings. These values are approximated by a constant for the considered frequency range. The values for the six coils and amplifier channels used in this study are given in Table 1.

**Figure 6:**
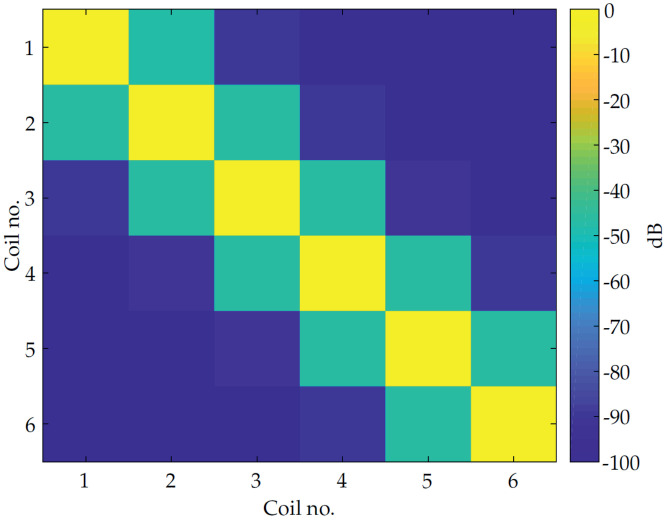
Cross correlation between individual equalized coil excitation signals.

The equalized signals are calculated according to

(10) $$x_{\mathrm{eq},i}\left(n\right)\;=\;g_{\mathrm{eq},i}\;\underset{{\tilde{x}}_{\mathrm{eq},i}\left(n\right)}{\underset{︸}{x_{\mathrm{ex},i}\left(n\right)*h_{\mathrm{eq},i}\left(n\right)}}$$

and forwarded to the matched filter. The weighting factor $g_{\mathrm{eq},i}=\frac{1}{E_{i}}$ ensures that each equalized signal has the same correlation output value one between the signals $x_{\mathrm{eq},i}\left(n\right)$ and ${\tilde{x}}_{\mathrm{eq},i}\left(n\right)$ at lag zero. The factor $E_{i}$ is the auto-correlation output of ${\tilde{x}}_{\mathrm{eq},i}\left(n\right)$ at lag zero. In Figure 6 the cross correlation of the signals $x_{\mathrm{eq},i}\left(n\right)$ and ${\tilde{x}}_{\mathrm{eq},i}\left(n\right)$ at time lag zero is visualized.

It is obvious that adjacent coils signals have cross correlation values different than zero. This is due to the decay behavior of the sensor impulse response. Nevertheless, the shown values are still sufficient for separating the coil signals.

### 4.2. Matched Filter

As described in Equation (2), the input signal of the sensor is a superposition of the magnetic coil signals. Additionally, noise sources superimpose the signal. To obtain a high signal-to-noise ratio (SNR) the coil amplitudes can be increased, which leads to a high energy consumption. Alternatively, a matched filter [27] can be used, which increases the SNR and thus can perform well also with lower energy consumption. This additionally makes the algorithm more robust against distortions. Hence, to obtain the amplitudes of the coil signals measured by the magnetoelectric sensor, the sensor input signal $x_{\mathrm{in}}\left(n\right)$ is matched filtered with the equalized coil excitation signals

(11) $$x_{\mathrm{mf},i}\left(n\right)\;=\;x_{\mathrm{in}}\left(n\right)*\;x_{\mathrm{eq},i}\left(L_{r}-n\right)\;.$$

The matched filter output can be evaluated every $L_{r}$ samples, since all coil signals have then been completely transmitted once

(12) $$m_{i}\left(k\right)\;=\;x_{\mathrm{mf},i}\left(kL_{r}\right)\;,$$

with the value $m_{i}\left(k\right)$ corresponding to the amplitude of the coil signal *i* at the sensor. These amplitudes are summarized in the vector $m\left(k\right)={\left[m_{1}\left(k\right),m_{2}\left(k\right),\ldots ,m_{N_{c}}\left(k\right)\right]}^{T}$ and forwarded to the localization algorithm.

### 4.3. Localization Algorithm

For the estimation of the position and orientation of the sensor, the matched filter output vector m(k) is compared with the columns of the lead-field matrix A. As stated in Equation (4), each column $a_{j}$ describes the coil amplitudes that would be measured by the sensor (after being filtered by the matched filter), if the sensor would occupy the defined position and orientation pair described by $p_{j}$. To be more robust against gain uncertainties and to ensure comparability between the measured coil amplitudes and the lead-field matrix columns, the vectors are normalized to the respective absolute maximum value beforehand. The values for the cost function $c\left(k\right)\;=\;{\left[c_{1}\left(k\right),\ldots ,c_{j}\left(k\right),\ldots ,c_{N_{p}}\left(k\right)\right]}^{T}$ are calculated by [15]:

(13) $$c_{j}\left(k\right)=\sum_{i=1}^{N_{c}}{\left|\frac{a_{ij}}{\max\left\{|a_{j}|\right\}}-\frac{m_{i}\left(k\right)}{\max\left\{|m(k)|\right\}}\right|}^{2}\;\forall \;j\in \left\{1,\ldots ,N_{p}\right\}$$

The estimated sensor position and orientation is then given by the position-orientation pair of the forward problem with the minimum cost function value

(14) $$\hat{p}\left(k\right)\;=\;p_{l(k)}\;\;\mathrm{with}\;l\left(k\right)=\underset{j}{\mathrm{argmin}\;}c_{j}\left(k\right)$$

and can also only be determined every $L_{r}$ samples. It is obvious that localization errors occur due to the discretization of the localization area. If the sensor is not directly located on a defined grid point, the localization error will be at least the distance between the closest grid point and the sensor’s location. Unfortunately, even higher localization errors can occur in some forward model configurations, due to the shape of the cost function c(k). Further information can be found in Appendix A. To overcome this problem a higher resolution is required. However, this will increase the computational complexity dramatically and hence can endanger the real-time capability of the system. By increasing the resolution iteratively, the localization can be performed with high accuracy and a moderate increase in computational complexity.

**Figure 7:**
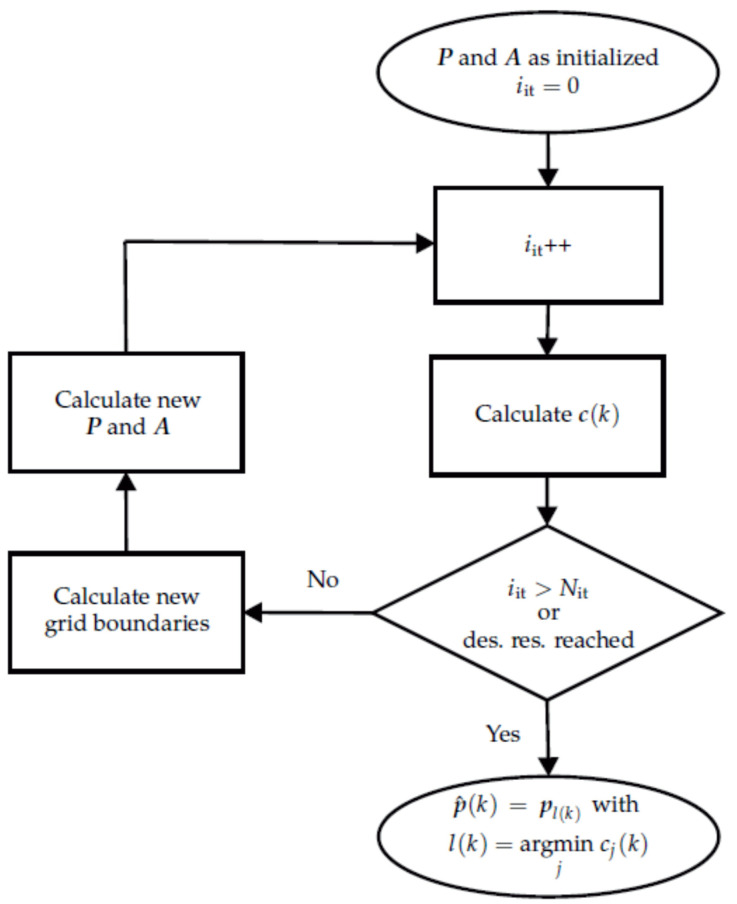
Flow chart of the iterative localization approach for one time step *k*. As long as neither the maximum number of iterations nor the desired resolution is reached, the algorithm keeps refining the localization grid.

The flow chart for the iterative localization process is shown in Figure 7. The first iteration is calculated as described before. Instead of considering only one estimated position-orientation-pair as stated in Equation (14), the $N_{b}$ best position-orientation-pairs are taken into account. The minima and maxima of the included position-orientation-pairs plus a fraction (one step size) of the previous grid form the boundaries of the new grid. The new grid is again divided into $N_{p}$ position-orientation-pairs and the forward problem as described in Equation (4) is calculated. From then on, the steps are repeated as described in the upper part of this section. Grid refinement stops when either the resolution between two adjacent grid points is less than a specified resolution or the maximum number of iterations $N_{\mathrm{it}}$ has been reached. The estimated position-orientation pair is given in the last iteration as described by Equation (14).

### 4.4. Postprocessing

To mitigate possible outliers in the localization results, a linear Kalman filter for smoothing the estimated localization outcomes is used. The measurement equation of the system can be described by

(15) $$\hat{p}\left(k\right)=Hs\left(k\right)+n_{m}\left(k\right),$$

with the state variables s(k), the observation model H transforming the states into the measurement variables, and supposing white Gaussian measurement noise $n_{m}\left(k\right)$ [28]. Assuming a linear system, the state variables are updated via the equation

(16) $$s\left(k\right)=Fs\left(k-1\right)+n_{p}\left(k\right)\;.$$

The matrix F is the state transition matrix and $n_{p}\left(k\right)$ is white noise with zero mean [28]. Due to the noise processes the measurement variables are subject to errors. The Kalman filter attempts to predict the states and thus reduces the influence of the noises stated in Equations (15) and (16). The calculations are performed according to the descriptions in [28]. Based on the $N_{m}=6$ measured variables summarized in $\hat{p}\left(k\right)$, there are $N_{s}=3\cdot N_{m}=18$ states available, when additionally considering the velocity and acceleration of the measured variables. The initialization of the variables and covariance matrices are described in Appendix B. The enhanced localization output is denoted as ${\hat{p}}^{\mathrm{enh}}\left(k\right)$. It is worth noting that the Kalman filter outputs should not lie outside the localization area and are thus restricted to the boundaries of the localization grid.

## 5. Measurements and Results

**Figure 8:**
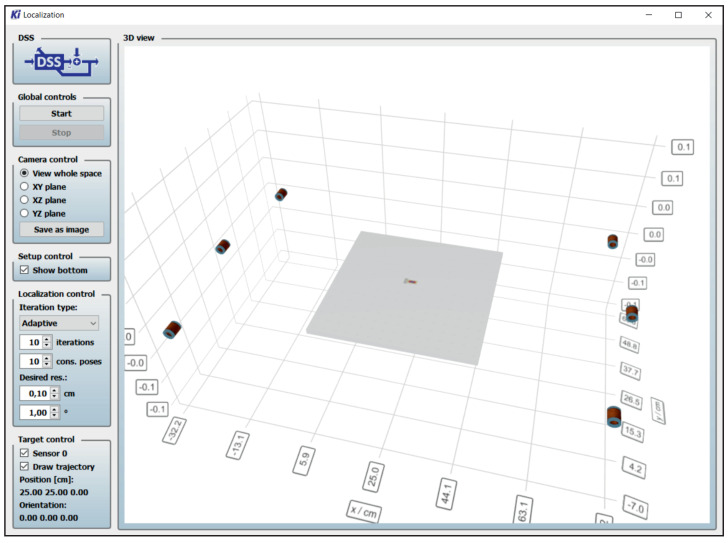
Graphical user interface of the real-time system used for localizing the magnetic sensors. The estimated position and orientation of the sensor is shown graphically in the 3D view and as text in the lower left corner. The number of iterations $N_{\mathrm{it}}$, the number of considered position-orientation pairs for refining the localization grid $N_{b}$ and the desired resolution in position and orientation are adjustable during runtime.

For the proof of principle, the measurements were performed in a two-dimensional space, i.e., only considering the x and y components of the sensor position and only considering the angle yaw ψ for the orientation of the sensor. The z component of the sensor’s position, as well as the orientation angles roll φ and pitch θ are assumed to be zero. Nevertheless, the proposed method can easily be performed in the three-dimensional space without any restrictions. More coils should be used for this, positioned in the three-dimensional space. Additionally, a smaller initial grid resolution might be used to keep the computational complexity low. The measurements were performed with a real-time system developed at the chair of Digital Signal Processing in Kiel [29]. A picture of the graphical user interface of the tool is shown in Figure 8.

The parameter used for the measurements (as defined in the sections above) are listed in Table 2. The localization area is limited to values between 0 cm and 50 cm in x and y direction and between $-90^{{}^{\circ}}$ and $90^{{}^{\circ}}$ for the yaw angle.

**Figure 9:**
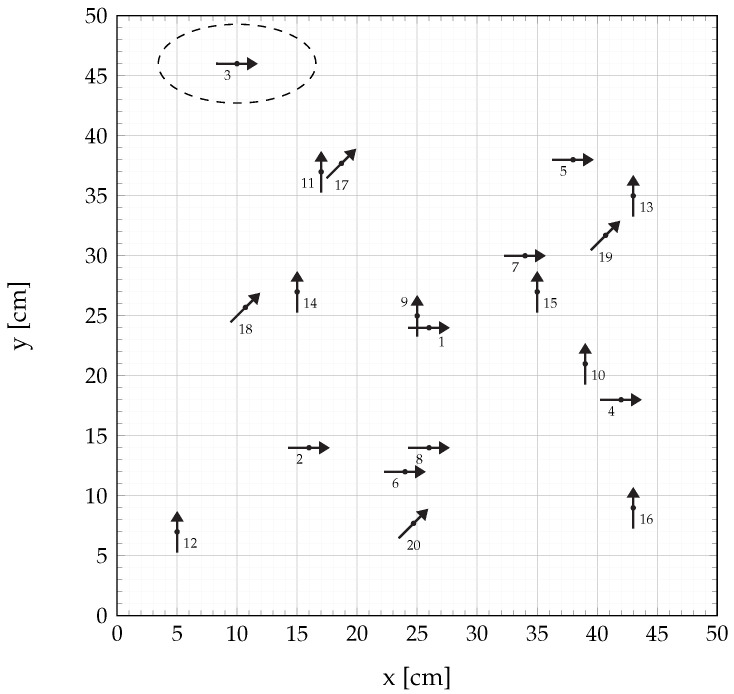
Position-orientation-pairs of the sensor used for testing the localization algorithm.

The waiting time between two consecutive coil signals must be rather high due to the decay of the impulse response of the sensor. Consequently, a total time of $\frac{L_{r}}{f_{s}}=896\;\mathrm{ms}$, with $f_{s}$ being the sampling rate, is needed to completely transmit the signals and thus to generate one localization result. This time can be shortened tremendously when using a sensor with a higher bandwidth. The sensor is placed in fixed positions and with fixed orientations to determine the accuracy of the algorithm. The tested position-orientation pairs $p_{s,j}$ were chosen randomly and are shown in Figure 9. The arrow directions represent the sensor’s long axis.

The tilt between the sensor’s sensitive and long axis has been approximately determined by manually rotating the sensor in a Helmholtz coil. The tilt was measured outside a shielded environment and results in $\gamma \approx -45^{{}^{\circ}}$. However, the tilt of the sensor depends on various factors, such as the strength of the bias field [30] (e.g., the earth’s magnetic field) and is therefore only an approximation.

**Figure 10:**
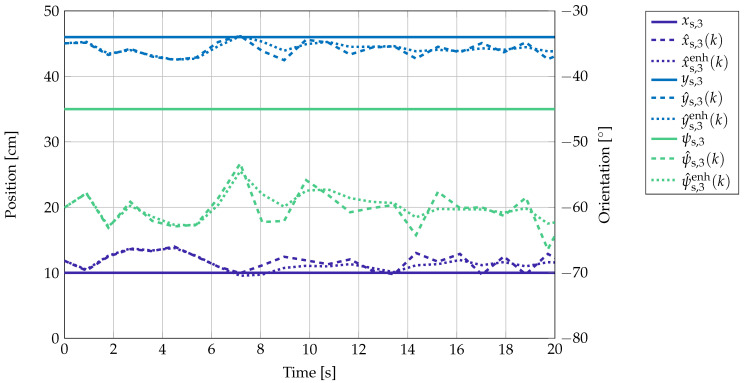
Real and estimated position and orientation of $p_{s,3}$ over time. The variances in the localization result are due to the presence of noise and cross talk in the measurement hardware.

The results of the localization for the position-orientation pair $p_{s,3}$ over time are shown as an example in Figure 10. Here, $x_{s,j}$, $y_{s,j}$, and $\psi_{s,j}$ denote the x and y component and the yaw angle of the sensor at position $p_{s,j}$, respectively. The localization output without the Kalman filter is described by ${\hat{x}}_{s,j}\left(k\right)$, ${\hat{y}}_{s,j}\left(k\right)$, and ${\hat{\psi }}_{s,j}\left(k\right)$ and after smoothing by the Kalman filter by ${\hat{x}}_{s,j}^{\mathrm{enh}}\left(k\right)$, ${\hat{y}}_{s,j}^{\mathrm{enh}}\left(k\right)$, and ${\hat{\psi }}_{s,j}^{\mathrm{enh}}\left(k\right)$.

There are some variances of the localization result over time. This is mainly due to the presence of noise, which leads to slightly varying amplitudes at the sensor and thus to small variations in the localization outcomes. The offset error might be due to cross talk between the coil amplifier channels and coupling of the magnetic coil signals into the cables and electronics. Additionally, it can be seen that the Kalman filter smooths the estimation output over time, so that outliers in the localization results do not have such a high impact.

**Figure 11:**
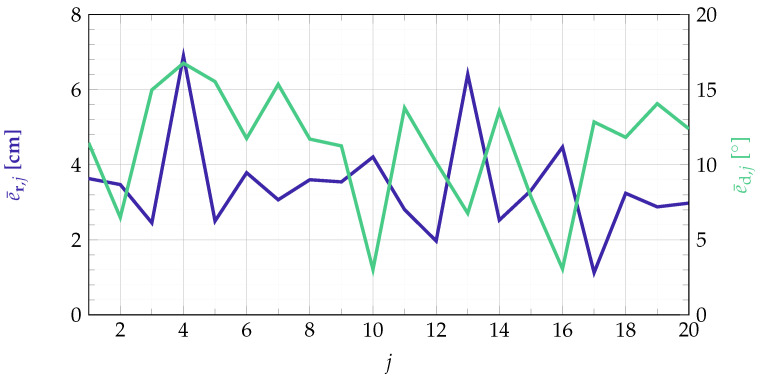
Mean localization errors for all tested position-orientation pairs. The index *j* depicts the respective position-orientation pair $p_{s,j}$ of the sensor as defined in Figure 9.

To quantify the accuracy of the algorithm, a localization error is defined according to

(17) $${\bar{e}}_{r,j}\;=\;\frac{1}{N_{\mathrm{meas}}}\sum_{k=0}^{N_{\mathrm{meas}}-1}\sqrt{{\left({\hat{x}}_{s,j}^{\mathrm{enh}}\left(k\right)-x_{s,j}\right)}^{2}+{\left({\hat{y}}_{s,j}^{\mathrm{enh}}\left(k\right)-y_{s,j}\right)}^{2}}$$

for the position estimation and

(18) $${\bar{e}}_{d,j}\;=\;\frac{1}{N_{\mathrm{meas}}}\sum_{k=0}^{N_{\mathrm{meas}}-1}\sqrt{{\left({\hat{\psi }}_{s,j}^{\mathrm{enh}}\left(k\right)-\psi_{s,j}\right)}^{2}}$$

for the orientation estimation. The number of localization outcomes is set to $N_{\mathrm{meas}}=50$, according to a measurement time of 44.8 s. The accuracy of the localization results for all tested position-orientation-pairs is shown in Figure 11.

The localization error is lying between 1.12 cm and 6.90 cm for the position estimation and results in a mean error of about 3.44 cm. The error for the orientation estimation is between 3.02${}^{{}^{\circ}}$ and 16.76${}^{{}^{\circ}}$. This results in a mean error of about 11.23${}^{{}^{\circ}}$. When considering fixed positions and orientations of the sensor, the localization output can be averaged over time and compared to the real sensor position/orientation afterwards. When doing so, the localization accuracy can be slightly improved and results in values between 0.46 cm and 6.52 cm for the position estimation and 1.54${}^{{}^{\circ}}$ and 15.35${}^{{}^{\circ}}$ for the orientation estimation. The average error reduces to 3.14 cm and 10.54${}^{{}^{\circ}}$, respectively.

The high errors for the estimation of the sensor’s position can result from the noise and the cross talk in the measurement system. Due to the different distances between the coils and the sensor the SNR is dependent on the sensor position/orientation. For example, looking at position $p_{s,9}$ an average SNR of about 9.5 dB is obtained at the sensor. Higher coil currents would lead to an improved SNR. Nevertheless, the goal was to localize with a minimum amount of energy. To avoid cross talk, the cables as well as the amplifier channels must be shielded. Additionally, the remaining cross talk can already be considered when setting up the forward problem or with an appropriate initial calibration. However, since the focus of this work is on the real-time localization pipeline and the calibration will be very extensive, it is not the subject of this work, but will be taken into account in our future work. The high error variance of the orientation estimation can partly be due to the change of the sensor’s sensitive axis with respect to the bias field. Even a rotation in the earth’s magnetic field can tilt the sensitive axis [30]. This problem only occurs with the sensors presented here and not with other types of magnetic field sensors. Furthermore, possible calibration errors do not only influence the position estimation but also the orientation estimation.

## 6. Conclusions and Outlook

An algorithmic pipeline for localizing magnetic sensors in real time was presented in this contribution. Besides the localization algorithm itself, pre- and postprocessing steps for an enhanced estimation of the sensor’s position and orientation have been described. The potential of the proposed algorithm was emphasized by measurements with a magnetoelectric exchange bias ΔE-effect sensor. Nevertheless, the proposed method can be applied to any type of magnetic field sensor. Only the coil excitation signals must be adapted to the properties (frequency range, dynamic range, etc.) of the magnetic sensor used. Using the magnetoelectric sensor, a mean localization error of 3.44 cm has been reached. For the proof of principle the localization of the sensor has been limited to the two-dimensional space. Nevertheless, the localization can be easily extended to the three-dimensional space.

The achieved results are comparable with other magnetic position estimation approaches. In [4] a 3×3m grid was used for localizing, achieving an accuracy of less than 10cm. Localizing with a 3D sensor in a grid size of 8×7cm an accuracy of 2.6mm could be reached in [31]. In [2] a localization accuracy of ≤2mm has been achieved, using coils for the localization of the sensors in a flexible MEG system. That shows that our localization method performs well, but can still be improved. However, the localization method investigated in this contribution can be performed in real time and in parallel to magnetic measurements without any degradation. Additionally, the robustness in noisy environments is increased by the usage of a matched filter and the smoothing of the localization outcomes via the linear Kalman filter. Moreover, the usage of a magnetoelectric sensor can be advantageous with respect to later medical applications due to its small size and the low production and operation costs.

Until now, the biggest limiting factor is the hardware used. Moreover, only a simplified model of the magnetoelectric sensor has been used, where the sensor is reduced to a point model. A more detailed model of the sensor, which also considers the dimensions of the sensor as well as a bias field dependent tilting of the sensor’s sensitive axis, could improve the localization accuracy. The results can be further improved using multiple sensors included in an array with fixed distances and orientations as described in [1]. To reduce the transmitting time of the coils and thus to increase the rate of localization outcomes, FDMA or CDMA approaches would be beneficial. This would require an adaption of the sensor hardware.

## Figures and Tables

**Table 1:** Parameter of the coils and amplifier channels used in this study. The conversion factors of the coils describing the relationship between current and magnetic field are described by the column *conversion*. The gains of the coil amplifier channels are normalized to the maximum value (channel 6). Both values are determined at the resonance frequency of the sensor.

Number	Conversion (mT/A) (@7712 Hz)	Amplifier Gain (Relative, @7712 Hz)
1	13.2	0.9632
2	12.4	0.9823
3	12.8	0.9592
4	12.8	0.9751
5	12.2	0.9549
6	12.5	1.0000

**Table 2:** Parameter of the coil signals used in this study.

Parameter	$N_{c}$	$L_{\mathrm{sig}}$	$L_{\mathrm{mf}}$	$f_{s}$ (kHz)	$N_{\mathrm{it}}$	$N_{b}$	$N_{p}$
**Value**	6	2048	28,672	192	10	10	49,419

**Figure A1:**
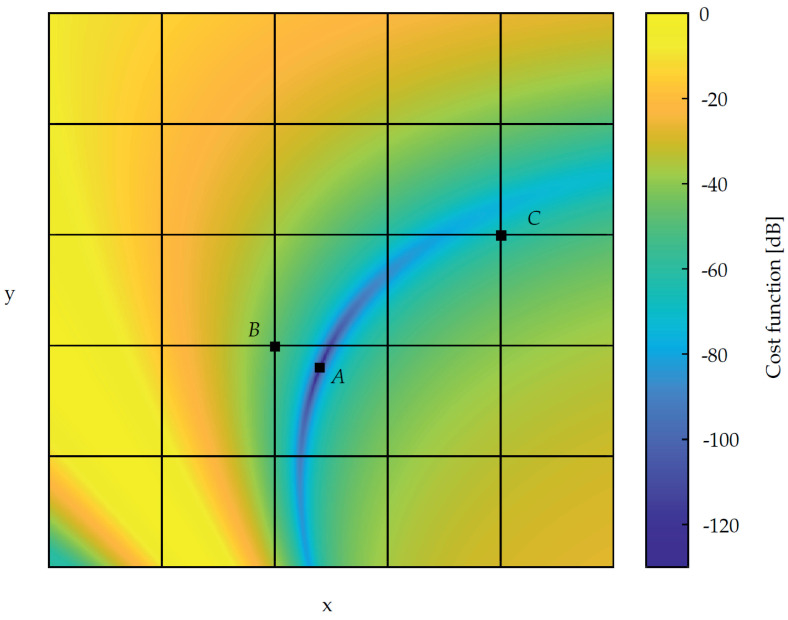
Exemplary cost function. The sensor is located at point *A*. Due to the relatively coarse grid (black lines), there will be a localization error of at least the distance between the point *B* and point *A*. However, due to the shape of the cost function, the minimum that is crossing the grid lines—and thus the localization outcome—is at point *C*.

## Data Availability

Not applicable.

## Abbreviations

The following abbreviations are used in this manuscript:

TDMA	Time Division Multiple Access
FDMA	Frequency Division Multiple Access
CDMA	Code Division Multiple Access
SNR	Signal-to-Noise Ratio

## Appendix A. Localization Errors

As already mentioned in Section 4.3, localization errors can occur due to the discretization of the localization area. It is obvious that the minimal localization error is defined between the real sensor position/orientation and the closest grid point and is only zero, when the sensor is lying directly on a defined grid point. Besides these obvious errors even higher localization errors can occur, if the grid resolution is not high enough. This highly depends on the coil arrangement, i.e., on the setup of the forward problem. In Figure A1 the cost function of a sensor located at point *A* (only considering a 2D plane and assuming a fixed orientation) is shown assuming an exemplary (not well set up) forward problem.

Now assuming a localization grid with a coarse resolution, which is visualized by the black lines. The points, where the lines are crossed are the potential grid positions. The minimal localization error would be the distance between point *A* and point *B*. However, the position-orientation pair with the smallest cost function is found at point *C*. This is due to the shape of the cost function and the small grid resolution, whereby the grid points do not lie on the minimum of the cost function. This results in a large localization error. Thus, a higher grid resolution is needed, which leads to a higher computational complexity and hence can endanger the real-time capability of the system. To overcome this, an iterative localization is implemented, which refines the grid resolution progressively.

## Appendix B. Initialization of the Kalman Matrices

As already stated in Section 4.4 the calculations of the Kalman filter are performed as described in [28]. The initialization of the matrices is filled as described for a discrete Wiener process acceleration model [28].

- State transition matrix
  (A1) $$F=\left[\begin{array}{ccc} I_{N_{m}} & \Delta tI_{N_{m}} & \frac{1}{2}\Delta t^{2}I_{N_{m}}\\ 0_{N_{m}\times N_{m}} & I_{N_{m}} & \Delta tI_{N_{m}}\\ 0_{N_{m}\times N_{m}} & 0_{N_{m}\times N_{m}} & I_{N_{m}} \end{array}\right]$$
- Measurement matrix
  (A2) $$H=\left[I_{N_{m}},0_{N_{m}\times 2N_{m}}\right]$$
- Covariance matrix of the process noise
  (A3) $$Q=E\left\{n_{p}\;n_{p}^{T}\right\}=\left[\begin{array}{ccc} \frac{\Delta t^{4}}{4}I_{N_{m}} & \frac{\Delta t^{3}}{2}I_{N_{m}} & \frac{\Delta t^{2}}{2}I_{N_{m}}\\ \frac{\Delta t^{3}}{2}I_{N_{m}} & \frac{\Delta t^{2}}{2}I_{N_{m}} & \Delta tI_{N_{m}}\\ \frac{\Delta t^{2}}{2}I_{N_{m}} & \Delta tI_{N_{m}} & I_{N_{m}} \end{array}\right]{\hat{\sigma }}_{p}^{2}$$
- Covariance matrix of the measurement noise
  (A4) $$R=E\left\{n_{m}\;n_{m}^{T}\right\}=I_{N_{m}}{\hat{\sigma }}_{m}^{2}$$
- State covariance matrix
  (A5) $$S\left(0\right)=I_{3N_{m}}{\hat{\sigma }}_{s}^{2}$$

Here, $I_{M_{1}}$ denotes a unit matrix of size $M_{1}\times M_{1}$ and $0_{M_{1}\times M_{2}}$ a matrix filled with zeros of size $M_{1}\times M_{2}$. E{…} is the expected value operator and $\Delta t=\frac{L_{r}}{f_{s}}$, with $f_{s}$ being the sampling rate. Considering the high measurement noise and assuming a slowly moving or fixed sensor (i.e., low process noise), the estimated variances are set to ${\hat{\sigma }}_{p}^{2}=0.001$, ${\hat{\sigma }}_{m}^{2}=10$, and ${\hat{\sigma }}_{s}^{2}=500$. These values were chosen exemplary.
